# An Experimental Test of Jealousy's Evolved Function: Imagined Partner Infidelity Induces Jealousy, Which Predicts Positive Attitude Towards Mate Retention

**DOI:** 10.1177/14747049241267226

**Published:** 2024-08-28

**Authors:** Steven Arnocky, Kayla Kubinec, Megan MacKinnon, Dwight Mazmanian

**Affiliations:** 1 Department of Psychology, 6057 Nipissing University, North Bay, Canada; 2 Department of Psychology, 7890 Lakehead University, Thunder Bay, Canada

**Keywords:** romantic jealousy, infidelity threat, mate retention, cost-inflicting mate retention, benefit provisioning mate retention, evolution of jealousy

## Abstract

Jealousy may have evolved to motivate adaptive compensatory behavior in response to threats to a valued relationship. This suggests that jealousy follows a temporal sequence: A perceived relational threat induces state feelings of jealousy which in turn motivates compensatory behavior, such as mate retention effort. Yet to date, tests of this mediation model have been limited to cross-sectional data. This study is the first to experimentally test this theoretical model. Men and women (*N *= 222) who were currently in committed romantic relationships were primed with an imagined partner infidelity (versus control) scenario. Participants then completed measures of state jealousy and intended mate retention behavior. Results found that those primed with the infidelity threat scenario experienced an increase in state jealousy, which in turn predicted more intended benefit-provisioning and cost-inflicting mate retention. Findings suggest that jealousy mediated the relationship between infidelity threat and intended mate retention behavior, supporting the evolutionary account of state jealousy.

## Introduction

Humans typically engage in biparental care of their young, who remain reliant on maternal and paternal investment for a protracted period of development (Trivers, 1972). This biparental care is facilitated by a mating system comprised of various forms of monogamy (e.g., serial, social, genetic) and long-term pair bonding that is rare among sexually reproducing species (Geary & Flinn, 2001). The formation of lengthy mating relationships introduces unique adaptive challenges, including the consistent threat of partner infidelity, which can diminish individuals’ reproductive success (Buss 1994, 1996). Some researchers have suggested that jealousy, a negative emotion characterized by feeling upset by a real or perceived threat to a valued relationship (Daly et al., 1982; Davis et al., 2018; Harris, 2004), evolved primarily to counteract the persistent threat of infidelity by motivating behavior, such as mate retention, to address that threat. Yet little research has tested this proposed model. The goal of this research was to experimentally examine whether priming romantically attached participants with a perceived threat of infidelity induces a state of jealousy, and whether that jealousy mediates the link between infidelity threat and subsequent positive attitudes toward performing mate retention behavior.

### The Evolutionary Perspective on Jealousy

From an evolutionary perspective, emotions are considered adaptations that respond to reliable challenges to survival or reproduction (ecological ‘inputs’; Buss, 2000; Tooby & Cosmides, 2016). When these challenges are detected, a signal is sent to activate the emotional program, evoking physiological responses manifested as motives or behaviors aimed at addressing the adaptive challenge (the ‘output’; Tooby & Cosmides, 2008). Inherent in this view is a clear temporal sequence. An environmental stimulus is perceived and processed by relevant cognitive-perceptual mechanisms, which in turn activate the appropriate emotional state. Emotion(s) then motivate appropriate behavior relevant to the initial evoking stimuli.

Some researchers have situated romantic jealousy within this broader evolutionary theory (see Davis et al., 2016 for review), as a basic human emotion with clearly evolved elements involving information processing, affect, and behavioral outputs (Buss, 2014). Daly et al. (1982) characterized jealousy as an emotion that is distinct from its associated behaviors, which functions as “a state that is aroused by a perceived threat to a valued relationship or position and motivates behavior aimed at countering the threat” (p. 12; see also: Tooby & Cosmides, 2008). Buss and Haselton (2005) similarly argue that “jealousy is an evolved adaptation, activated by threats to a valuable relationship, functioning to protect it from partial or total loss” (p. 506).

### Jealousy-Evoking (Cognitive/Perceptual) Inputs

Partner infidelity and defection pose serious adaptive challenges to men and women. Although sex differences exist in the sources and manifestations of jealousy along dimensions of sexual versus emotional types (Buss & Haselton, 2005), both sexes would have benefited most from being attuned to common underlying threats of a partner's infidelity or defection by expressing the general emotion of jealousy (Harris, 2004). Moreover, in ‘real life’ scenarios of suspected infidelity, the likely prospect of a partner's emotional and sexual infidelities being concurrent and fundamentally intertwined would make it difficult to parse apart their underlying contributions to the experience of jealousy. Indeed, both men and women are sensitive to cues of both kinds of infidelity (Shackelford & Buss, 1997), and most research examining overall levels of jealousy show no sex differences (see Madran, 2008).

### Jealousy-Evoked (Motivational/Behavioral) Outputs

Individuals sometimes engage in mate retention to counteract the adaptive challenges of partner infidelity or defection (Buss, 1988; Daly & Wilson, 1993). These behaviors can be classified into two categories: benefit-provisioning and cost-inflicting mate retention (Shackelford et al., 2005). Benefit-provisioning behavior confers benefits upon one's partner to entice them to stay in the relationship. Examples include gifts, complimenting one's partner, and public displays of affection (Miner et al., 2009). Alternatively, cost-inflicting behaviors can include actions that 1) impose a cost upon the partner, such as by lowering a partner's self-esteem or making them fearful to leave the relationship, or 2) harming or dissuading intrasexual rivals from mating with one's partner (McKibbin et al., 2007). Cost-inflicting tactics include monopolizing a partner's time, being overly vigilant about the partner's whereabouts, emotionally manipulating a partner, and intrasexual threats toward rivals (Holden et al., 2014). At its most extreme, cost inflicting mate retention can include emotional, verbal, physical, or sexual acts of intimate partner violence (IPV; Arnocky et al., 2015) or homicide (Kaighobadi et al., 2009).

The threat of infidelity is a consistent predictor of mate retention effort. Kaighobadi et al. (2008) found that men's accusations of female infidelity predicted their scores on the mate retention inventory. Moreover, men at greater risk of partner infidelity, such as those who have spent more time away from their partner since their last sexual encounter, engage in more mate retention effort (Starratt et al., 2007). Qualitative work has also suggested that in cases of men incarcerated for partner violence, telephone calls to their female victims suggest that accusations of infidelity often triggered their violent acts (Nemeth et al., 2012), and the severity of sexual and physical aggression experienced by abused women is greater among those who have had sex with other men (Shields & Hanneke, 1983).

The emotion of jealousy is also linked to mate retention effort (Naidin, 2022). Davis and colleagues found that cost-inflicting mate-retention was predicted by anxious (unease over potential infidelity) and preventive (preventing one's partner from consorting with others) jealousy, with the former also predicting benefit-provisioning mate retention. Those experiencing more social media (Facebook) jealousy score higher on overall mate retention and on digital acts of mate retention on social media (Brem et al., 2014). Beyond MRI scores, researchers have explored romantic jealousy in relation to more specific mate retention behaviors. Arnocky et al. (2023) showed that women higher in romantic jealousy held more positive attitudes and intentions toward costly and risky physical appearance enhancement, ostensibly as a form of benefit-provisioning mate retention. Puente and Cohen (2003) found that men who experienced jealousy perpetrated more emotional and sexual abuse against their partners. It has been suggested that abuse and restrictive behaviors of this nature may be a tactic to discourage partner infidelity and sexual competition (Daly et al., 1982). Ultimately, jealousy-induced mate retention might benefit individuals by reducing the threat of a partner's infidelity. For example, those with jealous partners report less perceived freedom to flirt with others, and this link was mediated by greater fear of their partner's reactions to their potential infidelities (Apostolou & Tzannetatou, 2023).

Nevertheless, little research has specifically examined whether jealousy mediates links between infidelity threat and mate retention attitudes, intentions, or behavior. Of the few studies examining this model, all have the major limitation of relying on cross-sectional data. For example, studies have found that women's romantic jealousy mediated the link between a relationship threat (perceiving oneself as being less attractive than same-sex rivals) and aggression toward partners and peers (Arnocky et al., 2012), as well as higher scores on the mate retention inventory (Arnocky & Locke, 2020). Among men in romantic relationships, anxiety (which has been characterized as an important component of jealousy; Buss, 1994) mediated the link between belief that their partner would commit infidelity and their partner violence perpetration (psychological, physical, sexual, and partner injury; Arnocky et al., 2015). Other research found that jealousy mediates the relationship between variables that could serve as proximate indicators of partner infidelity risk, such as anxious partner attachment, rejection sensitivity, or perceiving same-sex rivals as being more attractive in relation with mate retention (e.g., Arnocky & Locke, 2020; Murphy & Russell, 2018; Pakray & Dehshiri, 2023; Wright, 2017).

### The Present Study

The objective of the current study was to examine whether state jealousy mediates the link between an experimentally induced infidelity threat and intended mate retention behavior. We hypothesized that exposure to an infidelity threat condition (versus a negatively framed control condition) would induce a feeling of state jealousy (H1). We further expected that this induced state jealousy would mediate links to both intended benefit-provisioning and cost-inflicting mate retention over the next month (H2). Because infidelity poses an adaptive challenge to both men and women across the spectrum of adult romantic relationships, we expected these links to hold when controlling for both age and sex. However, because some sex differences have been identified surrounding the intensity and types of mate retention performed, we conducted an exploratory follow-up analysis to consider whether sex might moderate this mediation model.

## Method

### Participants

To determine an appropriate a priori sample size for mediation analyses, we followed guidelines set forth by Fritz and MacKinnon (2007). Based on established links between perceived partner infidelity to jealousy (Arnocky et al., 2015), and jealousy to mate retention (e.g., Davis et al., 2018; Degiuli et al., 2023), we expected small-to-medium effects for the a and b paths, yielding a minimum sample size of 148. We oversampled due to expected attrition from participant inattention or failure to adhere to instruction. A group of 739 potential respondents accessed the study using Amazon's Mechanical Turk. Only individuals who identified themselves as being in a current committed romantic relationship were included in the data set, determined using a demographic question asking their current relationship status. To be included in the final sample and receive compensation, participants also had to complete various attention checks. First, participants were denied if they spent less than the required time on the video or guided imagery priming tasks. Second, they had to correctly answer a question about the content of the video they were assigned to watch. Third, they had to correctly answer two randomly embedded attention check questions in the survey (i.e., “If you are paying attention to this survey, please select ‘Never’”). Participants with duplicate IP address were also removed. This resulted in a final sample of 222 participants (97 women) between the ages of 21 and 66 (*M *= 33, *SD *= 9.51). Participants were primarily heterosexual (75%) and Caucasian (96%, see Table 1). Sample demographics are presented in Table 1. Participants earned $1 USD for their time.

**Table 1.** Demographics.

Variable	N	Frequency (%)	M (SD)
Age (in years)	222		33.05 (9.51)
Sex	222		
Male	125	125 (56.3)	
Female	97	97 (43.7)	
Sexual Orientation	222		
Straight		163 (73.7)	
Gay/Lesbian		2 (0.9)	
Bisexual		57 (25.7)	
Ethnicity	222		
White/Caucasian		214 (96.4)	
Asian		2 (0.9)	
South Asian		2 (0.9)	
Black/African American		3 (1.4)	
Latin-American		1 (0.5)	
Condition	222		
Prime		104 (46.8)	
Control		118 (53.20)	

### Procedure and Measures

Participants were randomly assigned to one of two experimental conditions (infidelity priming task versus control). Participants then answered self-report measures in the order below.

#### Priming Task Versus Control

Participants were randomly assigned to either an infidelity threat condition or a control condition. Participants in the infidelity threat priming group were first directed to watch a one-minute TikTok video of a therapist highlighting how prevalent infidelity is in committed relationships (D’Arienzo, 2020). Participants in the control group first viewed a one-minute TikTok video of a negative food review (Bridgwater, 2022). We chose this video so that the control stimuli would also contain negative content unrelated to any relational or direct interpersonal functioning. Participant in both conditions then read a set of instructions for a guided imagery task:

You are now going to do a guided visualization task. Please read the instructions carefully and take as much time as you need to vividly imagine the scenario happening with as much detail as possible. Ensure that you spend at least 60 SECONDS engaging in the visualization task. Please click continue when you are ready to begin the visualization task.

The infidelity threat condition was based on previous research invoking imagined infidelity threat (Buss et al., 1992; Harris, 2000):

Now imagine that the person with whom you’re seriously involved becomes interested in someone else. Imagine you find out that your partner is having sex with this other person and is falling in love with that person. Take a moment, close your eyes, and try hard to visualize their infidelity happening, and feel the feelings you would have if this happened to you.

Those in the control condition engaged in the following guided imagery task:

Please think of a time you were excited for a meal. Imagine how much you looked forward to it. Now imagine that the meal you ate ended up being very disappointing. Imagine the food was poorly prepared with a bad taste. Take a moment, and try hard to visualize this happening, and feel the feelings you would have if this happened to you.

#### State Jealousy

A single-item state jealousy measure (Slotter et al., 2013) was used and slightly modified as follows, “At this moment, how jealous do you feel in your current romantic relationship?” Participants answered on a 7-point Likert-type scale ranging from 1 = *Not at all* to 7 = *Extremely*. This item was used to measure state feelings of jealousy and as a manipulation check between the control and prime group.

#### Mate Retention

Intended mate retention behaviors were measured using a modified version of the 38-item Mate Retention Inventory short form (Buss et al., 2008). Following others (e.g., Nascimento & Little, 2020), the short form of the MRI was modified to capture how often participants *intended* to engage in mate retention tactics in the following one month, using a 4-point Likert-type scale ranging from 1 = *Never* to 4 = *Often*. Example items are “Over the next month, I will display greater affection for my partner,” and “Over the next month, I will threaten to break up if my partner ever cheated on me.” Items were averaged to create mean scores for total mate retention as well as the two subscales. Total mate retention (*α* = .92), along with the Cost-inflicting (*α* = .89) and benefit-provisioning (*α* = .85) subscales showed good internal consistency.

## Results

### Analytic Plan

**Figure 1.**
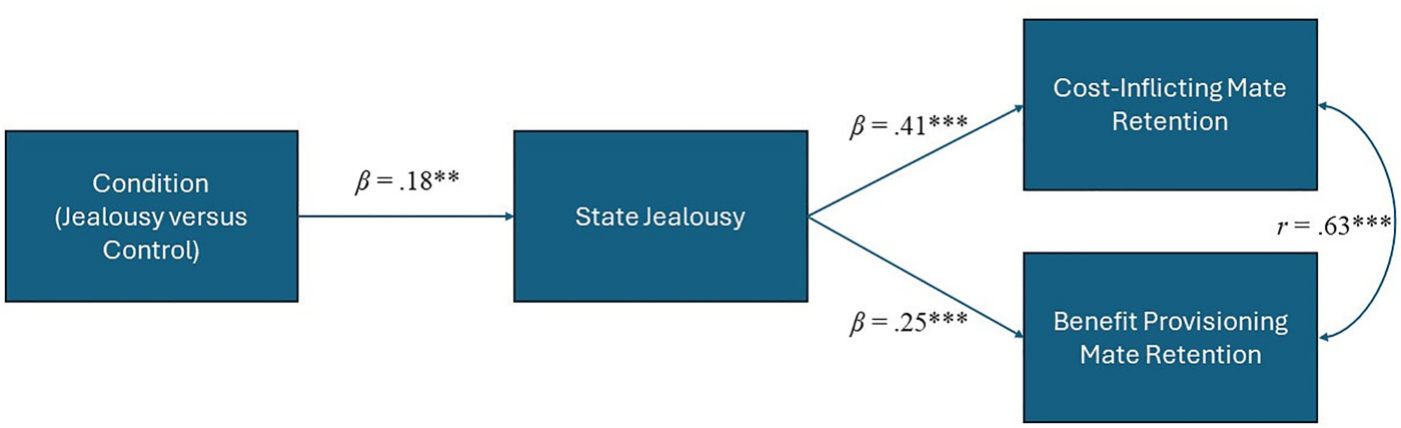
Indicators of statistical significance are as follows: **p < .01, ***p < .001. Sex and Age control variables not depicted for ease if visualization.

Path analysis via AMOS (version 29; Arbuckle, 2019) was used to explore whether exposing participants to an infidelity threat condition (versus control) would increase state jealousy, which we hypothesized would in turn predict both benefit-provisioning and cost-inflicting mate retention (Hypothesis 1), controlling for sex (male/female) and age (Figure 1). Model fit was assessed using the Chi-square test of significance (χ^2^), normed fit index (NFI), and the root mean square error of approximation (RMSEA; Kline, 2016). CFI values > 0.90, RMSEA values < 0.08, and a non-significant χ^2^ indicate adequate model fit. Indirect (mediation) effects were examined using 1000 bootstrap samples and bias-corrected 95% confidence intervals. Bootstrapping characterizes mediation not as the attenuation of the association between X and Y but the multiplication of the paths between X and M and M and Y (i.e., the indirect effect). Descriptive statistics and bivariate correlations are presented in Table 2.

**Table 2.** Descriptive Statistics and Bivariate Correlations for all Study Variables.

Variable	N	Mean	SD	Min.	Max.	1	2	3	4	5
1.Condition	222	—–	—–	—–	—–	—–				
2.Age	222	33.05	9.51	21	66	−.04	—–			
3.Sex	222	—–	—–	—–	—–	−.07	−.07	—–		
4.State Jealousy	222	5.49	1.33	1.00	7.00	.18**	−.10	.08	—–	
5.Benefit-Prov. MR	222	2.97	0.46	1.63	4.00	.10	−.19**	.09	.43***	—–
6.Cost-Inflict. MR	222	2.76	0.53	1.05	4.00	.03	−.06	−.05	.24***	.65***

*Note.* Indicators of statistical significance are as follows: **p < .01, ***p < .001.

First, we examined our mediation path model. Of the control variables, age correlated negatively with cost-inflicting mate retention (*r *= .16, *p *< .05). Sex was unrelated to both forms of mate retention, which were intercorrelated with one another (*r *= .63, *p *< .001). Results showed that the infidelity threat manipulation predicted greater reported state jealousy, relative to the control group (*b *= 0.49, *β *= .18, *SE *= .18, *p *< .01). State jealousy then predicted both benefit-provisioning, (*b *= 0.08, *β *= .25, *SE *= 02, *p *< .001) and cost-inflicting (*b *= 0.16, *β *= .41, *SE *= .02, *p *< .001) mate retention. The indirect effects showed that state jealousy mediated the link between the infidelity threat and both benefit-provisioning (*b *= 0.04, *β *= .05, *SE *= .02, *p *< .01, LLCI = .01 ULCI = .08) and cost-inflicting (*b *= 0.08, *β *= .08, *SE *= .03, *p *< .01, LLCI = .03 ULCI = .15) mate retention. The model fit the data well, *χ*^2 ^= 7.06 (*df *= 7, *p *= .42), RMSEA = 0.04 (95% CI = 0.00–0.087), NFI = .96.

Second, we conducted a follow-up moderated mediation analysis (PROCESS Model 14, Hayes, 2013) to explore whether sex (female = −.5, male = .5) moderated the paths between state jealousy and each form of mate retention, controlling for age (all mean-centered). Results showed that the jealousy x sex interaction was not statistically significant for either benefit-provisioning (*b *= 0.02, *t *= .43, *SE *= .04, *p *= .67) or cost-inflicting mate retention (*b *= 0.03, *t *= .71, *SE *= .05, *p *= .48). This suggests that the effects of induced state jealousy upon intended mate retention were not different across sex.

## Discussion

A key aspect of the evolutionary theory of jealousy is that it is an adaptive emotion that motivates compensatory behaviors (e.g., mate retention) when a threat to a valued relationship is perceived (e.g., infidelity threat). In contrast, others have argued that the cognitive/perceptual, emotional, and behavioral aspects of jealousy, whether normal or pathological, are experienced in a more simultaneous and interactive manner, in absence of any theoretically driven temporal sequence (Pfeiffer & Wong, 1989). Other researchers have argued that qualitative studies support social constructionist models (patriarchal social structure, threatened gender roles) and clinically relevant factors such as the ability to regulate emotion and resolve conflict, but not evolutionary models, in understanding “pathways” between infidelity, jealousy, and extreme cost-inflicting mate retention efforts (i.e., partner violence; Pichon et al., 2020). Although the evolutionary model does not explicitly suggest that these variables cannot interact with one another, it nevertheless demands evidence of the primary temporal sequence from perceived threat to emotion to behavior (acts or intentions).

We exposed individuals in committed romantic relationships to an infidelity threat (versus control) scenario to induce subsequent state jealousy, and then measured intended mate retention behavior. Results supported the hypothesis that imagined partner infidelity induced state jealousy, which in turn predicted more intended benefit-provisioning and cost-inflicting mate retention, regardless of participant age or sex. Our findings in support of the evolutionary model of jealousy are limited to the function of state jealousy, and do not address potentially different roles of pathological, morbid, or trait jealousy. Some theorists view more stable traits, such as morbid jealousy, as mutational noise in the originally adapted jealousy system (e.g., Tooby & Cosmides, 2008). Nevertheless, others have found that evolutionary informed sex differences in the experiences of pathological jealousy, such as in degree of upset over sexual versus emotional jealousy and in the rival attributes most likely to invoke jealousy, align with those observed in ‘normal’ jealousy (Easton et al., 2007). It would be interesting to examine whether exposure to infidelity threat information invokes a stronger emotional response and subsequent mate retention intentions among those who are higher in trait jealousy, given that they may express heightened sensitivity to infidelity threats. This could be explored by measuring jealousy at an earlier testing point, and then seeing whether this variable moderates the mediation model tested herein.

Our findings held regardless of participant sex. Previous research has identified some sex differences in the frequency of some mate retention behaviors. Nevertheless, it appears that induced jealousy more broadly activates a broader willingness to engage in both cost-inflicting and benefit-provisioning mate retention. This supports other research suggesting that overall, men and women report engaging in both broad strategies with similar frequency; albeit they may differ in some of the specific behaviors engaged in within these categories, such as appearance enhancement versus displays of resources.

These findings provide a stronger degree of evidence for the evolutionary theory of jealousy, relative to extant cross-sectional studies. Previous correlational research has explored this model, finding support that jealousy broadly mediates links between various relationship threats and a range of mate retention behaviors (e.g., Arnocky et al., 2012; Arnocky & Locke, 2020; Wright, 2017). Yet such cross-sectional studies are inherently limited in their efficacy to test temporally driven models. For example, although jealousy is believed to become activated when a reproductive threat is perceived, one can easily imagine the opposite to be plausible: That a jealous individual might be more prone to perceiving threats to the relationship, perhaps even as a component of their jealousy (e.g., Pfeiffer & Wong, 1989). The experimental jealousy induction addresses this issue of bidirectionality.

### Limitations and Future Directions

We used an online-survey format with a sample collected from Mechanical Turk. Researchers have raised concerns about data quality from such online sources (e.g., Nayak & Narayan, 2019). Other researchers have highlighted that Mturk participants are just as attentive, trusting of researchers, and buy in to interactive experimental procedures as much as with alternative convenience sampling techniques (Thomas & Clifford, 2017). We used a rigorous set of inclusion/exclusion criteria involving timing the priming task completion and embedded attention checks, which resulted in nearly 70% of those accessing the survey being rejected. Even with a remaining sample that appeared highly attentive, it is unclear how representative the study sample is of the broader population. Although it appeared heterogenous with respect to some demographic variables such as sexual orientation, other factors such as ethnicity were quite homogenous. Accordingly, future research should attempt to study links between jealousy and mate retention in broader cross-cultural populations. We also did not control incidences of past infidelity or past perpetration of infidelity in our sample. Those who have previously experienced infidelity may have different reactions to cues of infidelity. It has been found that those who have experience with previous partner infidelity are more suspicious of future partners’ behavior, and thus are more sensitive to cues of infidelity (Knopp et al., 2017). Our measure of a being in a committed romantic relationship was self-determined by the participant. This may leave room for variance in levels of commitment. We also did not screen for length or type of relationship (dating, marriage, etc.). Previous studies have found that length of marriage can be associated with a decrease in jealousy (Güçlü et al., 2017). Although random assignment across conditions should ensure an even distribution of relationship qualities across conditions, this would be worth measuring and controlling in future studies.

Our infidelity scenario encompassed both sexual and emotional infidelity. Due to established sex differences in the degree to which individuals are upset by the prospect or occurrence of sexual versus emotional infidelity (Buss & Haselton, 2005), research priming these threats in isolation might identify potential sex differences in the experience of state jealousy and the intended mate retention tactics that may follow.

Another limitation is that the overall emotional salience of both priming videos will never be identical. A cheating partner is likely more generally negatively arousing than a bad meal because it has more meaningful and longstanding consequences for one's life. It is therefore possible that the experimental and control condition stimuli may have differentially triggered other emotions besides jealousy. Because jealousy might conceivably co-occur with other emotions, it would be useful for future research to assess a wider range of emotions following exposure to the stimuli in order to better isolate the effects of any induced differences in jealousy. This would have the added benefit of allowing researchers to examine the roles of related emotions, such as anxiety (Arnocky et al., 2015), shown to relate to both infidelity threat and mate retention.

Another important limitation is the use of intended mate retention as the dependent variable. Of course, it is difficult to track actual mate retention following an experimentally induced infidelity threat. However, future research could consider a paradigm whereby the participant would attend a testing session with their partner. Following the threat condition used in this study, participants could be afforded the opportunity to allow or prevent their partner from engaging in a social task with an attractive confederate as a form of guarding, or they could be provided with the opportunity to spend their remuneration on a gift for themselves or their partner (e.g., benefit provisioning). Given recent research suggesting that jealousy-induced negative reactions might limit partners’ perceived freedom to flirt (Apostolou & Tzannetatou, 2023), it might also be interesting to examine perceived efficacy of mate retention tactics following infidelity priming. It is possible that those primed with partner infidelity threats would view various mate retention tactics as effective ways of preventing infidelity.

## Conclusion

Researchers have argued that jealousy was selected for in our species because it coordinated adaptive behavioral responses to perceived threats to valued relationships. In this study, participants exposed to an experimental infidelity threat condition reported higher state jealousy scores than those in the control condition. Jealousy, in turn, predicted more intended benefit-provisioning and cost-inflicting mate retention to be performed over the following month. These findings, which extend beyond extant cross-sectional tests of this model, support the perspective that jealousy plays a crucial role in responding to threats to mating relationships by motivating greater mate retention efforts.
